# Management of Uncomplicated Malaria in Underfives in Private and Public Health Facilities in South-Eastern Nigeria: A Clinical Audit of Current Practices

**DOI:** 10.1155/2013/575080

**Published:** 2013-01-21

**Authors:** Ekong Udoh, Angela Oyo-ita, Friday Odey, Emmanuel Effa, Ekpereonne Esu, Olabisi Oduwole, Moriam Chibuzor, Martin Meremikwu

**Affiliations:** Calabar Institute of Tropical Diseases Research and Prevention, University of Calabar Teaching Hospital, P.O. Box 1211, Calabar, Nigeria

## Abstract

Malaria remains a leading cause of underfive morbidity and mortality in sub-Saharan Africa. Effective case management is a strategy recommended by the World Health Organization for its control. A clinical audit of case management of uncomplicated malaria in underfives in health facilities in Cross River State, Nigeria, was conducted from January to March 2012. Data was extracted from patients' case records by trained medical personnel using pretested data extraction forms. Of the 463 case records reviewed, age, gender, and weight were reported in 98.1%, 97.3%, and 49.7% of the children, respectively. A history of fever was obtained in 89.6% and a record of temperature in 74.1% of the children. General examination was performed in 203 (43.8%) children. Malaria parasite test was requested in 132 (28.5%) while Packed cell volume or haemoglobin was requested in 107 (23.1%) children. Appropriate dose of Artemisinin Combination Therapy (ACT) was instituted in 300 (64.8%), wrong dose in 109 (23.5%), and inappropriate treatment in 41 (8.9%). The utilization of ACTs for treating uncomplicated malaria in the State has improved but clinical assessment of patients and laboratory confirmation of diagnosis are suboptimum.

## 1. Background

Malaria remains a leading cause of childhood illness and death in sub-Saharan Africa with an underfive annual mortality of approximately a million [1]. It is the most significant public health problem in Nigeria where it accounts for 25% of underfive mortality and 30% of childhood mortality. About 50% of the population will have at least one episode of malaria annually while about 24 million underfives will have 2 to 4 attacks of malaria annually [2].

Effective case management of uncomplicated malaria is a major strategy for malaria control. This entails proper clinical assessment, laboratory confirmation of the disease either by light microscopy or rapid diagnostic technique (RDT) prior to treatment with an effective antimalarial [3]. *Plasmodium falciparum *(*P. falciparum*) is the most virulent of all the species that infect humans and accounts for over 95% of malaria-related morbidity and mortality in the country [4]. Chloroquine (CQ) and Sulfadoxine-pyrimethamine (SP) were used as first-and second-line treatment, respectively, for uncomplicated malaria until the emergence and intensification of parasite resistance to these drugs which necessitated a review of the antimalarial treatment policy [5, 6]. The Nigerian government changed the treatment policy for uncomplicated malaria to the Artemisinin Combination Therapy (ACT) in 2005 in keeping with the WHO recommendation. This was based on reports of a national antimalarial therapeutic efficacy study conducted the previous year in the different parts of the country that showed Artemether-lumefantrine and Artesunate-amodiaquine to be more efficacious than CQ or SP in treating uncomplicated *P. falciparum* malaria infection in underfives [7]. The Nigerian government in partnership with some Non-Governmental Organizations has made significant progress in scaling up the procurement and distribution of RDTs and ACTs to health facilities in the country [8].

Most cases of malaria in the country are treated in private health facilities [9]. Effective case management of malaria in the private and public health facilities will not only reduce malaria-related underfive morbidity and mortality but will also prevent the emergence of parasite resistance to the ACTs [3]. A systematic assessment of case management of malaria in health facilities in comparison with standard clinical guidelines is therefore necessary.

The first clinical audit of case management of uncomplicated malaria in the region was conducted in 2006 just after the country officially transited from CQ or SP monotherapy to ACT as first-line treatment for uncomplicated malaria [10]. This was before the policy on laboratory confirmation of diagnosis prior to treatment was introduced by WHO in 2010 [3]. Changes in treatment guidelines such as this further give credence to periodic clinical audit of case management of uncomplicated malaria in health facilities in comparison with national and international treatment guidelines.

This clinical audit aimed at determining the extent to which the case management of uncomplicated malaria in underfives in Cross River State conforms to standard practice, identifying areas of shortfall and making recommendations to the Cross River State Ministry of Health on ways of improving the quality of care to underfives being managed for uncomplicated malaria in health facilities in the State. 

## 2. Methods

### 2.1. Design of the Study

This was a clinical audit on treatment of uncomplicated malaria conducted on the medical records of health facilities in Cross River State, Nigeria, between January and March 2012. 

### 2.2. Setting of the Study

The audit was conducted on medical records of both public and private health facilities in Cross River State. In the public sector, both secondary and primary health facilities were assessed, while, in the private sector, private hospitals and clinics were assessed. Facilities located in both urban and rural areas were assessed. For the purpose of this audit, private hospitals/clinics were categorized as secondary health facilities. 

Cross River State is one of the 36 political administrative states in the Federal Republic of Nigeria. Located in the south-eastern axis of the country within the tropical rain forest belt, the State has an annual rainfall of over 3500 millimetres. Malaria transmission is intense and perennial in this area.

### 2.3. Target Population

The target population for the study was children aged less than five years receiving care at primary and secondary health facilities in Cross River State in the preceding 3–6 months. Only underfives diagnosed and treated for uncomplicated malaria were included in this audit. Those above five years and underfives treated for severe malaria or childhood illnesses other than uncomplicated malaria were excluded. 

### 2.4. Sampling Methods

Cross River State is comprised of three senatorial districts each made up of 6-7 Local Government Areas (LGAs). A LGA was selected in each senatorial district by simple random sampling. The public primary health facilities were selected by simple random sampling from a list of health facilities in the State obtained from the Cross River State Ministry of Health. Five primary facilities (primary health centres) and five secondary facilities (1 general hospital and 4 private hospitals) were selected per LGA. For private hospitals, there was no comprehensive list of health facilities across the State. Thus, outside Calabar metropolis, the five biggest private facilities were selected based on the assumption that more information will be obtained from them for the audit. Records of patients treated for malaria as diagnosed by the clinician 3–6 months prior to the study were audited.

The target was to audit five primary health facilities in each of the 3 selected LGAs. In each facility, a putative number of 15 case records of underfives treated for uncomplicated malaria were randomly selected and audited. Likewise for each secondary (public and private) facility, we randomly selected and reviewed 15 records of underfives treated for uncomplicated malaria per facility. This number of case records was considered adequate by the investigators to reflect the current practice in the case management of malaria in underfives in health facilities in the State.

### 2.5. Tool for Data Collection

A clinical audit tool was developed from the monitoring and evaluation tools of the National Policy for Treatment of Malaria in Nigeria [11]. This tool was pretested and used to retrieve data from a sample of General Outpatient/Inpatient records of the selected facilities. Trained field workers (medical doctors, nurses, medical laboratory scientists, and community health officers) were responsible for extracting the data from hospital case record forms. 

### 2.6. Ethical Issues

The proposal for the audit was reviewed and approved by the Cross River State Health Research Ethics Committee. Consent to audit malaria treatment records of both the public and private hospitals/clinics was also sought and obtained from the Heads of those facilities. The confidentiality of the patients' record and clinicians' identity were adequately protected using identification numbers and codes for the patients and clinicians, respectively. The data extraction forms were archived in a cabinet under lock. 

### 2.7. Data Extraction

Information extracted from the records included patient's age, sex, weight, clinical features, diagnostic tests, and antimalarial drugs prescribed. The selected audit criteria in the data extraction form were checked as “Yes,” “NO,” or “Unclear” based on findings. “Laboratory test ordered” was classified when the attending clinician wrote down the tests to be done. “Laboratory tests done” was classified when the results were recorded in the patient's record of the result sheets. The laboratory tests considered in this audit were those of malaria diagnosis (confirmed either by light microscopy or RDT) and haematocrit (PCV) or haemoglobin (Hb) estimation. Children with PCV ≤ 21 g/dL were classified as severely anaemic [12, 13]. Patients' age and weight were used to determine the appropriateness of the dosage of drug administered. This was compared with standard tables obtained from the national malaria treatment guidelines. Treatment regimen was classified either as appropriate (correct dose of ACT), inappropriate (under or overdose of ACT), wrong treatment (drug not approved for malaria), or unable to determine. 

### 2.8. Data Management

Data entry and analysis were done with Microsoft Excel 2007.

## 3. Results

### 3.1. General Characteristics of the Children

A total of 463 case records of underfives managed for uncomplicated malaria in 30 health facilities across the State were audited. Of this number, weight was recorded in 230 (49.7%) and temperature in 343 (74.1%) as shown in Table 1.

### 3.2. Clinical Features of the Children

The history of fever was obtained in about 89.6%, while general examination was performed in 43.8% of the children. General examination was performed more frequently in the private facilities as shown in Table 2.

### 3.3. Laboratory Investigations and Results of the Children

Malaria test was requested for in 132 (28.5%) of the cases reviewed. Light microscopy was the main laboratory diagnostic method in 114 (86.4%). High level parasitaemia (+++/numerous) was recorded in 24 (5.3%) children, all of whom presented at the private facilities. Haemoglobin or PCV was requested in 107 (23.1%) of all children seen and was performed in 95 (88.8%) of them. Of the 95 children that had PCV estimation, 13 (13.7%) had severe anaemia all of whom presented at the private health facilities as shown in Table 3. 

### 3.4. Category of Treatment Obtained

A total of 300 (64.8%) cases were treated with an Artemisinin-based Combination Therapy (ACT). About 74% of children managed in the public facilities had an appropriate treatment regimen for uncomplicated malaria as against 42.2% in the private health facilities. Wrong dosage prescription was noted in 109 (23.5%). This was twice more likely in the private setting than in the public. The antimalarial prescription was inappropriate in 41 (8.9%) of the children treated in the facilities as shown in Table 4.

The inappropriate drugs prescribed in the private health facilities were limited to Chloroquine (CQ) and Artesunate as monotherapy, while the public health facilities had a wide range of medications. CQ constituted 56.1% of the total inappropriate prescriptions. This was followed by Sulfadoxine-pyrimethamine in 22.04% and inappropriate use of antibiotics in 9.8% of the cases.

## 4. Discussion

The findings of this practice audit show that the overall management of uncomplicated malaria in underfives in these institutions are inconsistent with the recommendations of the treatment guidelines of the national malaria control programme and that of the World Health Organization [3, 14]. While age was well documented in most cases, records of the patients' weight were available for only 50% of the cases reviewed.

Since weight and age are required to determine the dose of the recommended first-line treatment for Artemether-lumefantrine and other ACTs, failing to record or use these parameters would invariably lead to the use of inappropriate dosage of these drugs. The current ACTs deployed in the country have predetermined doses matched for weight band and age range inscribed on the packets. This has simplified the determination of correct dose of antimalarial and improved prescription pattern among health care providers in the absence of weight measurement. This is a laudable innovation given that a high proportion of children seen in health facilities in developing countries are often not weighed before being treated as observed in this audit. In low-resource settings where instruments for measurements are unavailable, obtaining the necessary demographic information could be helpful in the decision-making process in patient management.

History of fever (89.6%) or temperature measurement (74.1%) was recorded in a higher proportion of patients than weight. There is much room for improvement in this area since history of fever or measured axillary temperature ≥37.5°C is crucial for the definition of symptomatic malaria in children [3]. There was a paucity of information on the symptoms of malaria in the case records of patients seen in both facilities. Some of the missing information may be among the defining criteria for severe malaria and could help to determine the presence or absence of concomitant infections. For example, pallor, enlarged liver, and spleen which are often present in uncomplicated malaria were reported in relatively smaller proportion of patients. While hepatomegaly and splenomegaly may not have immediate diagnostic and prognostic significance in uncomplicated malaria, the presence of either or both of these could prompt further investigations that may reveal such concurrent illness as sickle cell disorder or septicaemia [15, 16]. The low prevalence of these clinical signs in this study reflects the failure of the clinicians to adequately examine the patients for them. Another Nigerian study reported that inadequate evaluation and underreporting of clinical findings were more common in the private than public health facilities [17]. The reverse is the case in the present study. Health providers in public health facilities tend to receive more training support from government agencies than their counterparts in private facilities and may be expected to adhere better to practice guidelines, but this does not appear to be the case in this audit [18]. It seems likely that a higher patient load in public health facilities makes such adherence difficult.

Most children were treated without parasitological confirmation of diagnosis which is a practice that is now being discouraged. The Nigeria National Malaria Treatment Policy Guidelines has recently been revised making parasitological test confirmation mandatory for diagnosis and treatment of malaria in all age groups [14]. The low rate of laboratory diagnosis of malaria in this study is similar to a report from a hospital-based clinical audit in a children's ward in Malawi [19]. The public health facilities were more likely to initiate treatment without laboratory confirmation of diagnosis than the private facilities. It is worthy of note that a high proportion of the diagnosis was done using light microscopy with most patients having low grade parasitaemia. The use of RDTs was low despite the fact that this is currently being promoted as an acceptable alternative to microscopy where the latter is not available or feasible [3, 14]. This may indicate a short supply of the commodity in the facilities since its use does not require much technical expertise when compared with light microscopy. Six of the children managed in the private health facilities were reported to have had high parasite density. These children could probably have been wrongly classified as having uncomplicated malaria because hyperparasitaemia is one of the case-defining criteria for severe malaria with attendant increase in risk of mortality in underfives [3]. Such misclassification of severity of illness as may have been the case in this audit could lead to wrong treatment and poor outcome.

Packed cell volume (PCV) or haemoglobin (Hb) was reported in less than a third of the cases reviewed. This was reported in one-third of the children managed in a health facility in Malawi [19]. The assessment of PCV or Hb in children being managed for malaria in the State is quite low. The high prevalence of *P. falciparum* infection in the country makes the underfives vulnerable to rapid progression in disease severity and potentially fatal complications. Since anaemia is a common complication of malaria in endemic regions [12, 20], it would be beneficial to assess underfives suspected of having malaria for anaemia by routinely measuring PCV or Hb.

Over 60% of the children had appropriate treatment regimen using an ACT. This finding indicates a marked improvement in the utilization of ACTs for treating uncomplicated falciparum malaria when compared with 3% recorded in the State just after the country formally transited from Chloroquine (CQ) or Sulfadoxine-pyrimethamine (SP) monotherapy to ACTs [10]. A similar increase in ACT utilization was observed in the Federal Capital Territory (FCT) of Nigeria where its prescription for treatment of uncomplicated malaria was as high as 81.4% [21]. This study, however, shows that a reasonable proportion of the doses of ACT prescribed were wrong. Wrong dosage of drugs was more common in the private health facilities than in the public health facilities. This could partly be due to the fact that most of the children in those facilities were not weighed before treatment was instituted. Wrong dosage of ACTs, especially subtherapeutic doses, will not only lead to treatment failure but could result in drug pressure and subsequent development of parasite resistance to the drugs. Indeed, reduced efficacy of artemisinins and ACTs has already been reported in some parts of Asia [22, 23]. Since the ACTs are currently the first-line treatment for uncomplicated malaria, effort needs to be made to prevent them from being wrongly prescribed. Prescription of subtherapeutic doses of CQ has shown to have contributed to the emergence and intensification of parasite resistance to the drug [17]. Wrong or irrational ACT prescription should therefore be checked to prevent the development of parasite resistance to this useful drug.

About 9% of the children were treated with drugs that are inappropriate for uncomplicated malaria. This is also a remarkable improvement from earlier Nigeria studies that reported higher use of CQ and SP [24, 25]. CQ remains the leading inappropriate antimalarial used in the health facilities in the treatment of uncomplicated malaria. Further public enlightenment, training, and retraining of health providers on the national malaria treatment guideline would enhance rational use of antimalarial drugs among all cadres of health care workers. 

## 5. Conclusions

The utilization of ACTs as a first-line therapy for uncomplicated malaria has significantly improved. However, there is a need to improve clinical evaluation and laboratory confirmation of malaria diagnosis in health facilities as recommended by the national and international malaria treatment guidelines. More efforts should be made to encourage and support health facilities to use RDTs for the diagnosis of malaria where light microscopy is not readily available or feasible.

## Figures and Tables

**Table 1:** Record of general characteristics of children.

General characteristics of patients	Number of patients (%)		
	Public	Private	Total
	328 (70.8%)	135 (29.2%)	463
Record of age:			
Yes	327 (99.7%)	127 (94.1%)	454 (98.1%)
No	1	8	9
Record of gender:			
Yes	309 (94.2%)	125 (92.6%)	434 (97.3%)
No	19	10	29
Record of weight:			
Yes	179 (54.6%)	51 (37.8%)	230 (49.7%)
No	149	84	233
Record of temperature:			
Yes	258 (78.7%)	85 (63.0%)	343 (74.1%)
No	70	50	120

**Table 2:** Record of clinical features reported in the children.

History/presenting symptoms	Public	Private	Total
	328	135	463
Fever	292 (89.0%)	123 (91.1%)	415 (89.6%)
Cough	175 (53.4%)	68 (50.4%)	243 (52.5%)
Vomiting	76 (23.2%)	41 (30.4%)	117 (25.3%)
Headache	10 (3.0%)	20 (14.8%)	30 (6.5%)
General examination done?			
Yes	99 (30.2%)	104 (77.0%)	203 (43.8%)
No	185 (56.4%)	30 (22.2%)	215 (46.4%)
Unclear	44 (13.4%)	1 (0.8%)	45 (9.7%)
Specific check for:			
Pallor	32 (9.8%)	55 (40.7%)	87 (18.8%)
Liver	16 (4.9%)	50 (37.0%)	66 (14.3%)
Spleen	21 (6.4%)	54 (40.0%)	75 (16.2%)

**Table 3:** Record of laboratory investigations and results.

	Public	Private	Total
	328	135	463
Malaria parasite test ordered?			
Yes	45 (13.7%)	87 (64.4%)	132 (28.5%)
No	283 (86.3%)	48 (35.6%)	331 (71.5%)
Malaria microscopy done?			
Yes	28/45 (62.2%)	86/87 (98.9%)	114/132 (86.4%)
No	17/45 (37.8%)	1/87 (1.1%)	18/132 (13.6%)
Malaria RDT done?			
Yes	12/45 (26.7%)	13/87 (14.9%)	25/132 (18.9%)
No	33/45 (73.3%)	74/87 (86.1%)	101/132 (76.5%)
% PCV/Hb ordered	24/328 (7.3%)	83/135 (61.5%)	107/463 (23.1%)
% PCV/Hb done	16/24 (66.7%)	79/83 (95.2%)	95/107 (88.8%)
Severe anaemia: PCV ≤ 21% or Hb ≤ 7 g/dL	0/16 (0%)	13/79 (17.3%)	13/95 (13.7%)

**Table 4:** Record of treatment administered for malaria.

Appropriateness of treatment given	Public	Private	Total
	328	135	463
Dosage and drug appropriate	243 (74.1%)	57 (42.2%)	300 (64.8%)
Drug appropriate but dosage wrong	59 (18.0%)	50 (37.0%)	109 (23.5%)
Drug not appropriate for malaria	25 (7.6%)	16 (11.9%)	41 (8.9%)
Unable to determine	1 (0.3%)	12 (8.9%)	13 (2.8%)
